# Enhancing the management of long COVID in general practice: a scoping review

**DOI:** 10.3399/BJGPO.2021.0178

**Published:** 2022-06-29

**Authors:** Aimee Brennan, John Broughan, Geoff McCombe, John Brennan, Claire Collins, Ronan Fawsitt, Joe Gallagher, Allys Guérandel, Brendan O'Kelly, Diarmuid Quinlan, John S Lambert, Walter Cullen

**Affiliations:** 1 School of Medicine, University College Dublin, Dublin, Ireland; 2 Royal College of Physicians of Ireland, Dublin, Ireland; 3 Irish College of General Practitioners, Dublin, Ireland; 4 Castle Gardens Surgery, Kilkenny, Ireland; 5 Ireland East Hospital Group, Dublin, Ireland; 6 Department of Psychiatry and Mental Health Research, St Vincent’s University Hospital, Dublin, Ireland; 7 Mater Misericordiae University Hospital, Dublin, Ireland; 8 The Rotunda Hospital, Dublin, Ireland

**Keywords:** COVID-19, delivery of health care, general practice, long COVID, post-acute COVID-19 syndrome, primary health care

## Abstract

**Background:**

Long COVID is a multifaceted condition, and it has impacted a considerable proportion of those with acute COVID-19. Affected patients often have complex care needs requiring holistic and multidisciplinary care, the kind routinely provided in general practice. However, there is limited evidence regarding GP interventions.

**Aim:**

This study aimed to identify key concepts and knowledge gaps around long COVID by conducting a scoping review of literature on the condition’s management by GPs.

**Design & setting:**

Arksey and O’Malley’s six-stage scoping review framework, with recommendations by Levac *et al*, was used.

**Method:**

PubMed, Google Scholar, the Cochrane Library, Scopus, and Google searches were conducted to identify relevant peer reviewed and grey literature, and study selection process was conducted according to the PRISMA Extension for Scoping Reviews guidelines. Braun and Clarke’s ‘Thematic Analysis’ approach was used to interpret data.

**Results:**

Nineteen of 972 identified articles were selected for review. These included peer reviewed articles and grey literature spanning a wide range of countries. Six themes were identified regarding GP management of long COVID, these being: (1) GP uncertainty, (2) listening and empathy, (3) assessment and monitoring of symptoms, (4) coordinating access to appropriate services, (5) facilitating provision of continual and integrated multidisciplinary care and (6) need to provide or facilitate psychological support.

**Conclusion:**

The findings show that GPs can play and have played a key role in the management of long COVID, and that patient care can be improved through better understanding of patient experiences, standardised approaches for symptom identification and treatment, and facilitation of access to multidisciplinary specialist services when needed. Future research evaluating focused GP interventions is needed.

## How this fits in

Knowledge around COVID-19’s long-term health impacts has grown, but little is known about the role that GPs can play in promoting affected patients’ outcomes. This study provides a comprehensive account of existing research around best practice for GP management of patients with long COVID. The findings indicate that patient care can be improved through better understanding of patient experiences, standardised approaches for symptom identification and treatment, and facilitation of access to multidisciplinary specialist services when needed.

## Introduction

A considerable proportion of people with acute COVID-19 remain unwell beyond 3 weeks, although the exact prevalence of ongoing issues among post-COVID-19 patients has not yet been firmly established.^1–3^ Knowledge gaps regarding the prevalence of long COVID are likely owing to several factors, perhaps notably the significant heterogeneity in long COVID definitions to date (for example, ‘post-acute COVID-19’, ‘chronic COVID-19’, ‘ongoing symptomatic COVID-19’, ‘post-COVID-19 syndrome’),^4,5^ and the fact that long COVID case recording practices are not commonly used within clinical settings, and certainly not to the same extent that acute COVID-19 cases are recorded.^5–8^

Common long COVID symptoms include fatigue, dyspnoea, chest pain, fever, palpitations, muscle and/or joint aches, metabolic disturbances, cognitive impairment, headaches, abdominal pain, new onset seizures, and dizziness.^6,7,9,10^ Patients report a substantial impact on quality of life and social functioning, with feelings of helplessness, uncertainty, fatigue, and inability to participate in normal activities contributing strongly.^11,12^

There is presently limited evidence and understanding concerning management of long COVID. Emerging research indicates that continuity of care is important owing to the condition’s ongoing nature, particularly with regard to support for and monitoring of symptoms.^13^ Further, the multifaceted nature of observed symptoms means that most patients can be managed in the community by holistic carers and multidisciplinary teams, with referral to specialty services when necessary.^9,14^ Integration of care systems is especially crucial to ensure that affected patients’ care needs are met.^15,16^

Thus, it has been claimed that primary care is well suited to managing patients with long COVID.^5,9,17^ However, research outlining the potential contributions of individual professions working in primary care is lacking, and this is very much the case regarding research around the role of GPs. This study aims to address this problem by reviewing the extant literature on best practice for GP management of long COVID. It is hoped that the study’s findings will serve as a useful resource for GPs seeking evidence-based guidance around care for affected patients.

## Method

Scoping review methods were chosen to establish an overview of emerging literature regarding GP management of long COVID, with an aim to identifying key concepts and knowledge gaps around the issue. The scoping review framework used comprises a six-stage process developed by Arksey and O’Malley^18^ with recommendations by Levac *et al*^19^ A protocol was not produced for the review.

### Step 1: Identifying the research question

The question 'How can GPs enhance the management of patients with long COVID in general practice?' was developed according to gaps in the literature, national (that is, Irish) and international health policy priorities,^20–22^ and the outcomes of consultation with clinical and research experts in general practice, infectious diseases, and psychiatry.

### Step 2: Identifying relevant articles

Initial database searching was undertaken on the 11 June 2021, and a reading list containing relevant articles was generated. This was followed by identification of keywords from the titles and abstracts of articles on the reading list, and the conducting of a second and more comprehensive database search using the identified keywords, which was completed on the 23 June 2021. The databases searched were PubMed, Google Scholar, the Cochrane Library, and Scopus. Google searches were also performed to identify relevant grey literature. Search terms were broadly grouped under headings relating to COVID-19 infection, persistent COVID-19, and general practice (see Box 1). The reference lists of relevant articles were manually searched to identify additional articles.

Box 1 **Search strategy**

((Post-acute COVID-19 syndrome OR long covid OR chronic covid syndrome OR long haul COVID OR long-haul COVID OR long hauler COVID OR post-acute sequelae of SARS-CoV-2 infection OR COVID-19 OR COVID19 OR COVID OR coronavirus disease 2019 OR COVID-19 Virus Disease OR SARS-CoV-2 Infection OR 2019 Novel Coronavirus Infection OR COVID*) AND (General pract* OR Family pract*))

### Step 3: Selecting articles

Initial searches and screening was conducted by one reviewer (AB). These search results were examined, duplicates were removed, and the following two levels of screening were conducted: a title and/or abstract review (AB); and full-text review. Full-text screening was performed independently by two reviewers (AB and JB), and any disagreements regarding study inclusion or exclusion were resolved through discussion by these reviewers. As is conventional with scoping reviews, inclusion criteria were broad, thus facilitating inclusion of both peer reviewed and grey literature. There were also no inclusion restrictions on research designs or degree of article quality.

Articles were included if:

1. They discussed management of long COVID in general practice, and
2. Their full text was available in English.

Articles were excluded if:

1. They did not discuss management of long COVID in general practice, and/or
2. Their full text was not available in English.

### Step 4: Charting data

Relevant data from included articles was organised into a table by AB and JB (see Supplementary Table S1) to facilitate analysis, and the data were charted according to the guidance of Arksey and O’Malley^18^ under the headings: Author, Year, Location, Research design, Title, Population, Definition(s) of persistent COVID-19 issues used, and Major findings.

### Step 5: Collating, summarising, and reporting results

Data were collated, presented, and reported by two independent reviewers (AB and JB) to ensure rigour, in line with Braun and Clarke’s thematic analysis approach.^23^ In accordance with Braun and Clarke’s guidelines, familiarisation with the data was gained via the study search and screening processes, codes relevant to the study’s aims were extracted and collated into themes using NVivo (version 12) software, and these themes were refined before composition of this study’s results section.

### Stage 6: Consultation

In line with Arksey and O’Malley and Levac *et al*’s recommendations,^18,19^ articles were also included and interpreted based on consultation with experts in general practice, infectious diseases, and psychiatry.^19^ Stakeholder consultation involved sharing study aims and working drafts with stakeholders for feedback purposes, and stakeholders provided ongoing consultation through email and online video-conferencing platforms.

## Results

**Figure 1.**
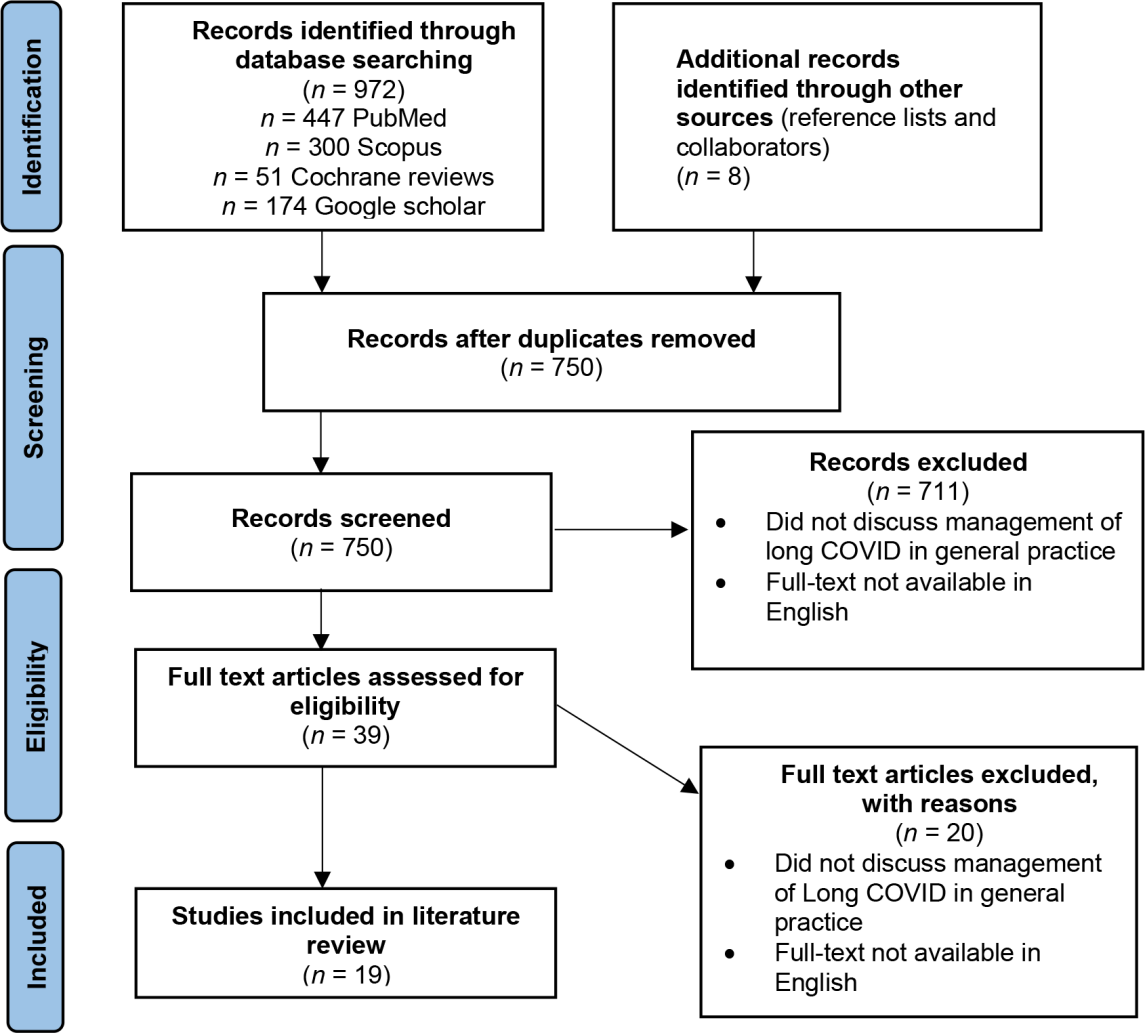
PRISMA extension for scoping reviews (PRISMA ScR) flowchart

Initial searching identified 972 articles. Eight more were identified following collaborator consultation and reference-list reviewing. Two hundred and thirty duplicates were removed, leaving 750 articles for screening. Seven hundred and eleven articles were excluded following title and/or abstract reviewing as they were not relevant to the research question. The remaining 39 articles underwent full-text review, and 19 were identified for inclusion. The PRISMA extension for scoping reviews (PRISMA ScR) flowchart outlines the study selection process (Figure 1).

### Description of included articles

Six articles were guideline documents delivered on behalf of prominent healthcare institutions in the UK, Canada, and Australia (for example, National Institute for Health and Care Excellence [NICE], NHS, and Royal College of General Practitioners [RCGP]);^15,16,24–27^ three were qualitative studies involving in-depth interviews and focus groups;^12,28,29^ two were editorials;^17,30^ two were case studies.^14,31^ There was one international policy brief,^22^ one cohort study,^32^ one pilot study,^33^ one ‘practice pointer’,^9^ one literature review,^34^ and one mixed-method study involving both survey and interview methods^35^ (see Table S1).

### Integrated findings

Thematic analysis of the included articles resulted in identification of the six themes outlined below.

#### Theme 1: GP uncertainty

Six articles documented GP uncertainty when managing patients with long COVID.^12,25,28–35,35^ Patients reported self-advocating because GPs were unsure how to investigate and manage symptoms.^28^ Meanwhile, GPs reported being uncertain on how to diagnose long COVID issues, a problem that largely stemmed from the fact that multiple persistent COVID-19 definitions have been proposed so far and from the multiplicity of long COVID symptoms, many of which overlap with other conditions that can arise separate to, or as a result of, COVID-19.^31^ GPs also described a lack of guidance when managing COVID-19 patients discharged from hospital, stating that the true level of need was only understood once patients were home.^35^ Further, GPs called for more guidance with regard to steering patients’ care and recovery,^25,29,35^ and stated that such guidance should be collated by and channelled through a single professional group or national body to avoid ‘guideline fatigue’.^35^

#### Theme 2: Listening and empathy

Four articles identified the need for GPs to listen to patients and demonstrate concern, empathy, and understanding.^12,25,28,30^ Patients emphasised the importance of being believed, listened to, and having their symptoms taken seriously.^12,28,30^ Patients expressed difficulty in finding GPs who believed their symptoms were real, and they voiced disappointment with a perceived lack of interest and support from the GPs they consulted.^12,28^ Patients also reported selectively disclosing symptoms to avoid symptoms being dismissed or negatively perceived by GPs.^28^

#### Theme 3: Assessment and monitoring of symptoms

Seven articles highlighted GPs’ roles in assessing and monitoring symptoms.^9,12,15,17,26,30,34^ Included articles indicated that GPs can guide initial assessments of physical and/or psychological functioning, and that GPs can play a key part with regard to excluding alternative diagnoses and serious complications.^15,17,30^ The articles also recommended that GP assessment and/or monitoring activities be pragmatic, person-centred, holistic, and continual.^15,26^ An NHS document advised that GPs assess and monitor people who experience new or persisting symptoms beyond 12 weeks,^15^ and it was also advised that GPs be mindful when assessing and monitoring vulnerable populations that have greater care needs (that is, people who tested positive, those who have been hospitalised for COVID-19, and patients with comorbidity).^32,33^ NHS England has recommended using three screening tools to assess symptoms, functioning, and care needs, namely: the Newcastle post-COVID syndrome Follow-up Screening Questionnaire; the COVID-19 Yorkshire Rehabilitation Screening tool; and the Post-COVID-19 Functional Status tool.^15^ Included articles also stated that GP software can also play a role in reliably coding for all relevant data categories to assist with monitoring, while also indicating that surveillance systems are needed to monitor complications and long-term illness.^12,15^

#### Theme 4: Coordinating access to appropriate services

Included articles stressed that care accessibility is important for affected patients and that GPs can guide navigation of referral pathways.^9,30^ Despite this, five articles described inadequate access to services for patients.^9,12,25,30,35^ The articles indicated that access to follow-up services needs improvement and that trusted guidelines should be translated into services.^30^ GPs reported finding referral to community follow-up services difficult and they expressed differing opinions about whose responsibility this was, as well as problems with waiting lists.^35^ One study showed that only 23% of survey responders had access to a post-COVID clinic, and a similar number reported having restricted access for patients who had not received a positive COVID-19 test result.^25^ Only 7% of survey responders felt they had good diagnostic testing access within the community.^25^ Lastly, patients have stated that they felt they were not entitled to access care owing to the invisibility of symptoms and experiences of not being taken seriously.^12^

#### Theme 5: Facilitating provision of continual and integrated multidisciplinary care

Eight articles described GPs’ roles in facilitating continual multidisciplinary patient care.^9,14–26,30,31,35,35^ Included articles indicated that most referrals to specialist services come from GPs and that strong links between GPs and specialist services are necessary to establish and maintain referral pathways.^9,16,26,31^ On patients’ return to GP care following hospital discharge, GPs have expressed a need for better communication between GPs and hospital teams.^14,35^ GPs have also described a need for improved follow-up services and reported believing that community teams, like those for stroke or cardiac rehabilitation, should be set up for patients discharged from the intensive care unit (ICU) after COVID-19.^35^ It has also been suggested that access to post-COVID clinics for all suitable patients should be more available following GP referral.^15^

#### Theme 6: Need to provide or facilitate psychological support

Four articles described the need for GPs to facilitate psychological support.^15,17,27,35^ GPs expressed concern about the complex psychological needs of patients recovering from severe COVID-19, and they stated that disproportionate emphasis has been placed on patients’ physical needs.^35^ Included articles indicated that new or recurrent psychiatric symptoms should be assessed and managed as per normal GP guidelines, and that GPs should screen for mood, anxiety, and substance use disorders, as well as post-traumatic stress disorder (PTSD), and ideation of homicide, self-harm, and/or suicide.^27^ It was noted that referral should be considered in line with existing local mental health pathways where a mental health condition has been identified as the predominant symptom,^15^ and that GPs can improve patients’ mental health by re-establishing social connections, encouraging community and peer support, and focusing attention on structural determinants of health (for example, poverty, discrimination, and social injustice).^17^ It was also said that GPs may offer reassurance and consider recommending non-pharmacological treatments including meditation, exercise, psychotherapy, and telemedicine resources.^27^ Lastly, GPs were advised to address physical symptoms that may indirectly contribute to poorer quality of life and subsequently adverse mental health and wellbeing.^27^

## Discussion

### Summary

This review aimed to map the emergent literature regarding GP management of long COVID. The findings indicate that GPs can, and often do, play a key role in the management of long COVID, and that long COVID care in general practice can be improved through better understanding of patient experiences, use of standardised approaches for symptom identification and treatment, and the facilitation of enhanced access to multidisciplinary specialist services for patients when needed.

### Strengths and limitations

Knowledge regarding the scale, nature, and management of long COVID is still emergent, and the exploratory nature of articles included in this review reflects this. The scoping review method used was therefore appropriate because scoping reviews are well suited to mapping literature around novel research areas that usually involve inductive knowledge acquisition approaches. The use of Arksey and O’Malley’s scoping review framework,^18^ with revisions by Levac *et al*,^19^ the PRISMA ScR framework,^36^ and Braun and Clarke’s thematic analysis approach^23^ were also beneficial, as they collectively ensured that the review’s research development, article selection, and data interpretation processes were conducted in a valid, transparent, and rigorous manner. However, this review’s method also had limitations that should be considered. While the authors tried to be comprehensive in their approach, it is possible that some relevant literature was not identified by the search terms or databases used. Moreover, as is conventional with scoping reviews, assessment of research quality was not conducted. Only articles published in English were considered, and this approach may have resulted in the exclusion of relevant literature. Lastly, there was no patient and public involvement (PPI) in the conduct of this review, an omission which may have adversely affected the study’s applicability to these groups’ experiences.^37^

### Comparison with existing literature

This study provides what is perhaps the most comprehensive account of research to date regarding GP management of long COVID. While an increasingly large number of published articles have examined care practices for affected patients,^4,6,9,11^ few of these (and to the authors' knowledge, no *reviews*) have focused as closely on the unique contributions that individual care professions can offer. This study’s key findings and recommendations also support several of those made in previous research on care provision for patients with long COVID and health issues more broadly. For instance, this study’s findings support previous claims that care providers (GPs and otherwise) may enhance patient outcomes by improving their understanding of patient experiences and perspectives,^38^ and by proactively availing themselves of appropriate symptom assessment and treatment strategies and referral pathways.^15,16,39^

### Implications for research and practice

First, it is worth acknowledging that COVID-19 has had considerable adverse impacts on general practice internationally, resulting in widespread reduction of capacity and quality of patient care,^40^ as well as increased GP workload and burnout.^41,42^ This study’s findings may bring some clarity to the management of long COVID in general practice, and so may warrant optimism in the general practice community. The findings may also inform patient care through encouraging greater listening and compassion for affected patients, an approach that aligns with person-centred care principles and one that can form a basis for partnership in co-producing better health outcomes.^43–45^ Furthermore, the findings may inform guidelines for symptom identification, diagnosis, treatment, and referral. These contributions may in turn help towards standardising ongoing care practices like those for other chronic conditions.^46^ Educational initiatives for GPs may also be helpful. Programmes aiming to promote GP understanding of patient perspectives may gain from PPI,^47^ and educational initiatives around best practice for diagnostic and referral procedures may be beneficial. Policymakers, meanwhile, are advised to support the implementation and trialling of recommendations in clinical settings. As mentioned, long COVID is a multifaceted condition requiring multidisciplinary care. Thus, policy support may involve providing structures and resources for the implementation of interventions not only within general practice, but also within wider health and social care systems, using an integrated care approach akin to Ireland’s Sláintecare model.^48^ As for future research, there is a need to develop and evaluate such interventions. This review’s findings suggest that research examining interventions targeting enhancement of doctor—patient relationships, symptom identification and treatment practices, and integrated care systems may have most value.
